# Student Aggression in a Military Medical University and Coping Strategies for Dealing With Stressful Conditions: A Cross‐Sectional Descriptive‐Analytical Study in Iran

**DOI:** 10.1002/hsr2.70899

**Published:** 2025-07-27

**Authors:** Mohammadreza Shabani, Haniyeh Meqdadipour, Fatemeh Kalroozi, Mohsen Moradi, Mohammad Hassan Kazemi Galougahi

**Affiliations:** ^1^ Student Research Committee, College of Nursing Aja University of Medical Sciences Tehran Iran; ^2^ Pediatric Nursing Department, College of Nursing Aja University of Medical Sciences Tehran Iran; ^3^ Public Health and Psychiatric Nursing, School of Nursing and Midwifery Shahrekord University of Medical Sciences Shahrekord Iran; ^4^ Department of Social Medicine, Faculty of Medicine Aja University of Medical Sciences Tehran Iran

**Keywords:** aggression, encounter, military medicine, strategies, stressful situations

## Abstract

**Background and Aims:**

Students in military environments often face unique pressures and stressors. This study aimed to assess the levels of aggression and the coping strategies employed under stressful conditions among students at Aja University of Medical Sciences in Tehran, Iran.

**Methods:**

This descriptive‐analytical study was conducted during the first half of 2023 on 314 students from a military university. A purposeful, criterion‐based sampling method was used. Data were collected using three standardized instruments: the Aggression Questionnaire by Eysenck and Glenn Wilson (1975), the Stress Coping Strategies Questionnaire by Endler and Parker (1990), and a demographic information questionnaire. Statistical analysis was performed using SPSS software (Version 21), with a significance level set at *p* < 0.05 for all tests.

**Results:**

The study revealed that over 12% of the students exhibited high levels of aggression. The majority of participants (61.5%) employed task‐oriented coping strategies. Statistically significant relationships were found between aggression and several variables, including gender, academic semester, concurrent employment, a history of depression, and the use of tobacco and alcohol. No significant associations were observed between aggression and factors such as age, marital status, field of study, place of residence, or history of acute or chronic illness. Regarding coping strategies, students primarily relied on task‐oriented coping (61.5%), followed by emotion‐oriented coping (24.8%) and avoidance‐oriented coping (10.2%). Task‐oriented coping was significantly associated with a history of antidepressant use. Emotion‐oriented coping correlated significantly with academic semester, a history of depression, chronic physical illness, and the use of alcohol or tobacco. Avoidance‐oriented coping was significantly associated with gender, place of residence, a history of depression, and the use of alcohol or tobacco.

**Conclusion:**

The average aggression score among students was moderate. Given the demanding military environment of their education, there is a clear need to address aggression through targeted interventions. Structured anger management workshops and training programs are recommended to mitigate aggressive behavior and promote healthier coping mechanisms.

## 1 Introduction

The World Health Organization (WHO) classifies aggression and aggressive behavior among the top 20 causes of human life loss, with increasing disability and erosion [1]. Worldwide, violence and aggression are among the most common antisocial and abusive behaviors, especially among individuals aged 15–44 [1], and modernization and advancement of human societies have added challenges to this issue. Young people in this age group, due to experiencing adolescence and youth, undergo sudden and extensive changes [2]. Being accepted to university and transitioning from high school to university is considered a major change in life for many young people [3]. This change is considered a source of stress for some students, leading to maladaptive responses in them [4]. It is estimated that 246 million students worldwide have experienced aggression at least once, and this traumatic experience can lead to numerous physical and psychological problems throughout their lives [5]. For this reason, investigating the phenomenon of aggression and its underlying factors among medical science students is of critical importance, as this group plays a pivotal role in delivering healthcare services to society through direct interactions with patients and their family caregivers [6]. Given that aggression can impair both physical and psychological well‐being, its presence among medical science students may disrupt healthcare delivery processes and compromise patient safety [7].

Medical science students as future architects of the healthcare system, in this field, bear a profound responsibility to safeguard the quality of medical services [8]. Aggression not only threatens their personal health but also undermines clinical performance, diminishes the efficacy of therapeutic interventions, and erodes public trust in the healthcare system [9]. Addressing this issue is a critical step toward preserving the professional well‐being of students and enhancing therapeutic outcomes for society.

On the other hand, medical science students are always exposed to high levels of stress and psychological tension due to their direct exposure to vital human issues, high volume of theoretical and practical courses, heavy ethical responsibilities, heavy work shifts, idealism (e.g., becoming a skilled and excellent doctor or nurse), and relatively low income [10]. In this regard, a study conducted by Al‐Shahrani et al. in 2023 showed that more than 85% of medical students experience moderate to severe stress [11]. Also, a meta‐analysis study published in 2023 showed that more than 42% of nursing students experience moderate to severe stress [12]. In addition to the mentioned points, studying medical sciences in military universities, compared to nonmilitary ones, involves unique challenges stemming from the combination of rigorous medical training and strict military regulations [13]. Furthermore, restrictions on personal freedoms and compulsory service obligations in military institutions result in significantly higher levels of stress and tension among medical students in military universities compared to their counterparts in nonmilitary institutions [14]. Since this stress ultimately threatens the health of the individual and subsequently leads to a decrease in the quality of patient care and an increase in medical errors. Therefore, identifying stressors and providing effective coping strategies to manage them should be a priority for medical education systems [15].

In this regard, Zhang in her study believes that Endler and Parker suggested that there are three mechanisms for coping with stress: task‐focused coping or problem‐focused coping, which involves an active approach to solving the problem; emotion‐oriented coping, which focuses on emotional reactions to the problem; and avoidance‐oriented coping, which involves escaping or avoiding the situation or problem by either withdrawing from society and others or engaging in a new activity [16]. According to Endler and Parker's coping model, emotion‐focused or emotion‐oriented and avoidance‐oriented strategies are identified as maladaptive strategies, while task‐focused styles are seen as adaptive styles for coping with stressors [17]. Numerous studies have been conducted to determine the stressors faced by students and how they cope with them. For example, a study in Taiwan on 500 students found that most of these students used stress responses such as addiction to mobile phones and the internet, social withdrawal, or, in some cases, verbal violence towards others, and these reactions decreased after educational interventions and behavioral therapy [18]. In a study in Iran, it was found that 11% of students had a high level of hopelessness, and their field of study, marital status, and whether they received social support were closely related to their aggressive behaviors [19]. The research group in this study conducted an in‐depth search on the prevalence of aggression among Iranian medical science students, especially those studying at military medical universities, but unfortunately, accurate statistics were not obtained. However, it is clear that most articles acknowledge the high prevalence of aggression in this age group [20, 21]. The aim of this study was to investigate the level of aggression and coping with stressful conditions among students of the Aja University of Medical Sciences in Tehran to possibly help reduce the occurrence of this problem and the associated costs.

## 2 Materials and Methods

### 2.1 Study Design and Setting

This cross‐sectional descriptive‐analytical study was conducted in the first half of 2023 on 314 students of the military‐affiliated faculties who were studying in the bachelor's and doctorate degrees of medicine (medicine, dentistry, nursing, and paramedical) of Aja University of Medical Sciences, Tehran, who were studying between academic semesters 1 and 6. The sampling method was classified based on the objective and in accordance with the entry criteria. If more than 10% of the questionnaire items were left unanswered by a sample, they were excluded from the analysis, and other samples meeting the entry criteria were included to reach the desired number. With a total of 1148 students at the Aja University of Medical Sciences, and using the Morgan Table, a sample size of 285 was calculated, considering a 10% potential dropout rate; the final sample size in this study was calculated as 314. For each faculty, the required number of samples was calculated proportionally to the number of students, and they were included in the study.

The instruments used in this study were the standard Aggression Questionnaire by Eysenck and Glenn Wilson [23], the Stress Coping Strategies Questionnaire by Endler and Parker [23], and a researcher‐made Questionnaire of demographic information.

The standard Aggression Questionnaire consists of 30 questions, with responses given on a Likert scale of yes, no, or unsure. This questionnaire has 21 positively worded questions and nine negatively worded questions. This means that for positively worded questions, a response of yes receives 2 points, no receives 0 points, and unsure receives 1 point. For negatively worded questions, a response of no receives 2 points, yes receives 0 points, and unsure receives 1 point. The maximum score on this questionnaire is 60, and the minimum is zero. A score of 25–35 indicates moderate aggression, while scores above or below these values indicate higher or lower levels of aggression, respectively [24]. To confirm the formal and content validity of the standard Aggression Questionnaire by Eysenck and Glenn Wilson [22] and the Stress Coping Strategies Questionnaire by Endler and Parker [23], the tools were given to 10 faculty members and experts in psychiatry and counseling psychology. Based on their opinions, potential corrections were made. To assess the reliability of the Aggression Questionnaire by Eysenck and Glenn Wilson [22], Cronbach's alpha coefficient was used, resulting in a value of 0.92. In Iran, Ghoreyshi Rad examined the reliability of the questionnaire and reported Cronbach's alpha coefficient of 0.74 [22].

The second tool used in this study was the Stress Coping Strategies Questionnaire by Endler and Parker [23], which categorizes coping behaviors into three main areas: (1) task‐oriented coping, which involves active confrontation with and management of the problem (16 questions). (2) Emotion‐oriented coping, which focuses on emotional responses to the problem (16 questions). (3) Avoidance‐oriented coping, which involves escaping or avoiding the problem (16 questions). This questionnaire consists of 48 questions with a 5‐point Likert scale response format. The response range for each question is from 1 to 5, and the minimum and maximum scores on this questionnaire are 16–80. Option one indicates that the respondent never engages in such behavior, while option five indicates that the respondent engages in such behavior very frequently. Options two, three, and four mean “rarely,” “sometimes,” and “often” respectively. Based on the respondents' answers to the questions, each behavior that receives a higher score is considered the individual's coping style. The reliability of the second tool, the Stress Coping Strategies Questionnaire by Endler and Parker [23], was also assessed in the same way, and Cronbach's alpha coefficient of 0.84 was calculated. The reliability of this questionnaire in Iran was also examined, and Cronbach's alpha coefficient of 0.82 was reported [23].

The third questionnaire in this study included questions related to the demographic characteristics of the samples, such as age, gender, marital status, academic semester, faculty of study, field of study, student's GPA in the previous semester, place of residence, having a job concurrent with being a student, depression or anxiety and other psychological disorders, and acute or chronic physical illness. The questionnaires were provided in person to the participants, after obtaining a written informed consent form from them Before distributing the questionnaire, a form was obtained in which students were assured that participation in this study would not result in any financial or educational harm to them and would not interfere with their academic process. Also, before sampling, the objectives of the study were explained to the students, and a commitment was given that the information from the questionnaires would be completely confidential. It was also emphasized that the research team would compensate for any potential financial harm. After obtaining written informed consent, the questionnaires were given to the students and collected on the same day.

### 2.2 Statistical Analysis

SPSS (Ver. 21) statistical software was utilized for data analysis. Descriptive statistics (including mean, standard deviation, and frequency) and to examine the relationship between research variables, an independent *t*‐test, analysis of variance (ANOVA) test, and *χ* ^2^ were used. Moreover, the significance level in all statistical tests was considered at less than 0.05.

### 2.3 Ethical Consideration

This study was approved by the ethics committee of Aja University of Medical Sciences with the ethics code of IR.AJAUMS.REC.1402.081. also, All authors have read and approved the final version of the manuscript had full access to all of the data in this study and takes complete responsibility for the integrity of the data and the accuracy of the data analysis. Also, a well‐written informed consent form was appropriately obtained from each of the participants. The study protocol conformed to the ethical guidelines of the 1975 Declaration of Helsinki.

## 3 Results

This study aimed to investigate the level of aggression and coping strategies among 314 students of the Military University of Medical Sciences in Tehran, the capital of Iran. The mean age of the students was 20.40 ± 1.47 years. Among the participants, 218 (69.4%) were male, and 308 (98.1%) were single. The largest number of participants in this study was medical students, with 136 individuals (43.3%). Additionally, 311 participants (99%) were living in student dormitories (Table 1).

**Table 1:** Demographic characteristics of students participating in this study.

Variables	Percent	Number
Gender		
Male	69.4	218
Female	30.6	96
Marital status		
Single	98.1	308
Married	1.9	6
Term		
1	44.9	141
2	38.5	121
3	14.3	45
4	1.27	4
5	12.4	19
6	4.1	13
Field of study		
Medicine	43.3	136
Nursing	23.6	74
Dentistry	9.6	30
Paramedicine	23.6	74
Place of residence		
Dormitory	99	311
The house	1	3
Having a job and being a student at the same time		
No	92.7	291
Yes	7.3	23
History of mental illness		
No	76.8	241
Yes	23.2	73
History of physical illness		
No	94.9	298
Yes	5.1	16
History of taking antidepressants and anti‐anxiety drugs		
No	95.2	299
Yes	4.8	15
History of smoking or alcohol consumption		
No	93.0	292
Yes	7.0	22

One of the objectives of this study was to examine the studied students in terms of aggression levels and aggressive behaviors. The mean score for aggression among students was 27.12 ± 7.85. It was found that 40 individuals (12.7%) had a high level of aggression, and nearly half of the sample (44.6%) exhibited moderate aggression. Furthermore, most students (193 individuals, 61.5%) used problem‐solving coping strategies (with a mean score of 61.18 ± 14.78) (Table 2). The Pearson correlation test revealed a significant correlation between the aggression of the studied students and their emotion‐oriented coping strategies (*p* = 0.001, *r* = 0.239) and Avoidance oriented coping strategies (*r* = 0.291, *p* = 0.001).

**Table 2:** The mean and standard deviation of the aggression score and strategies for dealing with stress in the studied students.

Variables	Mean	Standard deviation	Frequency (percent%)
Aggression score			
Total aggression score	27.12	7.855	—
Stress coping strategy			
Task oriented	61.18	14.78	193 (61.5)
Emotion oriented	49.42	18.04	78 (24.8)
Avoidance oriented	47.56	15.99	32 (10.2)

Table 3 consists of two parts; the first part indicates the statistical relationship between aggression and demographic variables. The results of independent *t*‐tests in this section showed a significant relationship between aggression and gender, employment status, and history of smoking and alcohol consumption. The results of the Analysis of variance (ANOVA) in this table also indicate a significant relationship between aggression and academic semester. In the second part of the table, the results of independent *t*‐tests demonstrate a significant relationship between a history of anxiety and depression medication use and problem‐solving coping strategies, as well as between a history of psychological and physical illnesses, and smoking and alcohol consumption, with emotion‐oriented coping strategies.

**Table 3:** The relationship between aggression and adjustment with demographic information of students in this study.

				Coping strategies for stress								
	Aggression			Task oriented			Emotion oriented			Avoidance oriented		
Variables	Mean	Standard deviation	*p* value	Mean	Standard deviation	*p* value	Mean	Standard deviation	*p* value	Mean	Standard deviation	*p* value
Gender												
Male	27.93	8.162	0.01*	61.76	14.719	0.31*	49.76	18.64	0.62*	49.54	16.69	0.001*
Female	25.37	6.87^a^	2.59^a^	59.91	14.93	1^a^	48.70	16.76	0.48^a^	43.38	13.57	3.34 a
Marital status												
Single	27.15	7.92	0.92*	61.07	14.84	0.22*	49.16	17.87	0.89*	47.59	16.07	0.75*
Married	27.50	1.0^a^	−0.08^a^	71.87	10.82	−1.71^a^	51.56	18.54	−1.34^a^	50.52	14.43	−0.31^a^
Term												
1	25.27	7.72	0.009**	61.86	15.11	0.42**	48.22	17.88	0.01**	15.39	1.48	0.159**
2	30.55	7.68	3.12^b^	56.38	14.91	0.996^b^	53.80	17.42	3.07^b^	16.00	2.74	1.60^b^
3	27.90	8.36		60.93	15.26		53.22	17.56		15.58	1.64	
4	27.44	7.67		64.06	13.50		39.16	21.08		15.87	4.40	
5	26.07	5.34		62.96	13.94		46.56	17.361		16.89	2.81	
6	29.77	7.01		61.06	10.91		41.40			20.28	6.41	
Field of study												
Medicine	28.35	8.60	0.07**	61.41	15.33	0.701**	49.03	14.12	0.55**	45.79	15.85	0.288**
Nursing	25.39	6.38	2.36^b^	61.34	16.28	0.474	51.98	18.43	0.697^b^	48.25	18.23	1.26^b^
Dentistry	26.93	7.86		58.17	11.37		48.75	19.51		46.92	12.22	
Paramedicine	26.16	7.11		61.86	13.47		47.78	15.47		50.33	15.15	
Place of residence												
Dormitory	27.03	7.83	0.15*	61.14	14.79	0.76*	49.38	18.09	0.45*	47.40	15.89	0.03*
The house	35.33	6.42	−2.20^a^	66.40	18.78	−0.39^a^	54.68	6.62	−1.1^a^	71.09	18.78	−2.1^a^
Having a job and being a student at the same time												
No	26.72	7.28	0.002*	61.19	14.88	0.93*	49.48	17.93	0.81*	47.27	15.74	0.26*
Yes	32.40	12.41	−3.16^a^	60.93	13.69	0.084^a^	48.33	20.61	0.24^a^	51.3	18.99	−1.23^a^
History of mental illness												
No	26.29	6.80	0.001*	61.17	14.61	0.98*	48	17.42	0.009*	46.39	14.94	0.04*
Yes	29.81	10.17	−3.25^a^	61.21	15.49	−0.02^a^	54.76	19.42	−2.69^a^	51.53	18.72	−2.04^a^
History of physical illness												
No	27.08	7.87	0.74*	60.98	14.65	0.40*	48.71	17.61	0.002*	47.43	16.03	0.54*
Yes	27.79	7.67	−0.33^a^	64.89	17.24	−0.862	64.54	21.03	−3.13^a^	50	15.68	−0.60^a^
History of taking antidepressants and anti‐anxiety drugs												
No	26.90	7.73	0.09*	60.80	14.32	0.04*	48.71	17.61	0.19*	47.50	15.72	0.79*
Yes	31.69	9.27	−1.83^a^	68.86	21.51	−2^a^	64.54	21.03	−1.28^a^	48.66	21.38	−0.262^a^
History of smoking or alcohol consumption												
No	26.69	7.669	0.003*	60.82	14.68	0.15*	47.86	17.132	0.001*	46.27	15.10	0.001*
Yes	33.11	8.18	−3.31^a^	66.17	15.76	−1.47^a^	70.62	16.98	−5.73^a^	65	17.83	−5.28^a^

a *t*‐statistic value was obtained with an ANOVA test.

b *f*‐statistic value was obtained with an ANOVA test.

* *p* value was obtained with an independent *t*‐test

** *p* value was obtained with an ANOVA test.

## 4 Discussion

This study was conducted with the aim of determining the level of aggression and coping strategies for stressful conditions among students of the Aja University of Medical Sciences in Tehran during the years 2022–2023. Given that physical and mental health is an ideal and highly important perspective for all healthcare systems, especially military healthcare centers, focusing on their psychological and emotional challenges is of paramount importance.

Among the challenges facing students, experiencing aggression during confrontations with stressful life challenges and a lack of knowledge about coping strategies against stressors are significant [25]. Aggression can manifest physically, verbally, or mentally. These behaviors, besides harming the target individual, can lead to mental disorders, reduced academic or occupational performance, depression, anxiety, or even physical and bodily consequences [22]. Various reasons contribute to the occurrence of aggression among students, including intrinsic factors such as gender, financial status, inability to manage emotions and control anger, and inappropriate lifestyle [26]. Extrinsic factors include societal and familial pressures, especially from peer groups, socio‐cultural differences, academic competitions, limited educational resources and facilities, and the influence of virtual space [27]. Björkqvist et al. acknowledged that male students exhibit more physical aggression than female students [28]. In our study, a statistically significant relationship was also observed between gender and aggression. Additionally, another study has shown that unemployment and a lack of sufficient income lead to aggression and violence among individuals [29]. In our study, a statistically significant relationship was found between aggression and employment status.

Hart and Burns also found in a study in Australia that the higher the alcohol consumption among students, the significantly higher the occurrence of aggressive and criminal behaviors. The results of their study align with the findings of our study [30]. Additionally, Ruchkin et al. in Russia, examined 2600 young people in 2023, concluded that a history of depression and increased levels of depression lead to higher levels of aggression and violent behaviors among youth, which is more common in boys than in young girls [31]. The results of our study also indicated a statistically significant relationship between a history of depression and aggression. Furthermore, Pakseresht et al. in a study of 280 students in Iran, found a significant correlation between the tendency to smoke and use drugs with the occurrence of aggression, which is consistent with the results of our study [32]. Here, no statistically significant relationship was found between the aggression of students and their place of residence (dormitory vs. non‐dormitory), which contradicts Alami et al., as their results suggested that dormitory students exhibited significantly higher levels of aggression than non‐dormitory students [33].

By examining the literature, we find that controlling aggression and creating intimate and respectful environments for all individuals, especially students, is of utmost importance, as university environments should be filled with mental and emotional tranquility. One way to control aggression and anger is to have effective coping strategies against stressful conditions. The results of this study showed that more than 60% of medical students used task‐oriented coping strategies. In this regard, the results of Alkhawaldeh et al. in Oman showed that most medical students used task‐oriented [34] coping strategies during academic and daily life challenges. On the other hand, the results of our study showed a statistically significant relationship between the use of avoidant coping strategies and students' gender and place of residence, which is contrary to the above study. Furthermore, the results of our study showed a significant relationship between the use of avoidant coping strategies and a history of mental illness, which is in line with the results of a study by Rehr and Nguyen, who showed that students with symptoms of mental illness were more likely to use avoidant coping strategies [35].

Another significant finding of the current study was the statistically significant relationship between emotion‐oriented coping strategies and the variable of academic semester. In this regard, Neufeld and Malin examined how medical students cope with stress and showed that medical students entering the clinical education setting in their sixth semester tend to use less effective coping strategies when facing daily life and academic challenges, relying more on emotion‐oriented coping strategies [36].

In our study, it was evident that emotion‐oriented coping strategies had a statistically significant relationship with a history of mental illness among students. In this context, Abdollahi et al. showed that the use of emotion‐oriented coping strategies had a significant relationship with depression among undergraduate students [37]. Another result of our study was the statistically significant relationship between the use of emotion‐oriented coping strategies and a history of physical illnesses [38]. In this regard, Casagrande et al. demonstrated that individuals with hypertension and cardiovascular diseases do not use appropriate coping strategies when facing stressful conditions [39]. Another finding of our study was the statistically significant relationship between alcohol and drug consumption and emotion‐oriented coping strategies. Opalach et al. showed that increased alcohol consumption among homeless individuals leads to increased use of emotion‐oriented coping strategies [39]. Similarly, the Azizi et al. study demonstrated that individuals who used heroin significantly rely more on emotion‐oriented coping strategies when faced with stressful conditions [40].

## 5 Conclusion

The results indicated that the level of aggression among Aja medical students was moderate, with the highest levels observed among medical students and those struggling with alcohol use. Contrary to expectations, while there was no statistically significant relationship between aggression scores of dormitory and non‐dormitory students, dormitory residents exhibited lower aggression compared to their non‐dormitory counterparts. This suggests that dormitory life, despite challenges such as separation from parents, fosters independence and better adaptation to daily life. Notably, a significant proportion of participants in this study (nearly 39%) employed inappropriate coping strategies to manage daily stressors. This underscores the critical responsibility of healthcare policymakers and authorities in every country to create conditions that nurture the development of healthy, competent students, thereby advancing the healthcare system. Therefore, it is recommended that the curricula for medical students—particularly military medical students—incorporate not only specialized academic training but also essential skills such as communication techniques, problem‐solving strategies, and stress management tools for navigating daily personal and professional challenges. Such measures could help reduce aggression among students while enhancing their cognitive abilities to effectively address stressful problems and challenges.

## 6 Study Limitation

Among the limitations of this study are its cross‐sectional nature and the fact that it was conducted entirely based on student responses, which can be affected by various factors such as interpersonal differences. Also, the results of this study are based on the results obtained at one university and may differ for other universities.

## Author Contributions

**Mohammadreza Shabani:** writing – review and editing, writing – original draft. **Haniyeh Meqdadipour:** writing – original draft, writing – review and editing. **Fatemeh Kalroozi:** supervision, writing – review and editing. **Mohsen Moradi:** validation. **Mohammad Hassan Kazemi Galougahi:** methodology and validation.

## Conflicts of Interest

The authors declare no conflicts of interest.

## Transparency Statement

The corresponding author, Fatemeh Kalroozi, affirms that this manuscript is an honest, accurate, and transparent account of the study being reported; that no important aspects of the study have been omitted; and that any discrepancies from the study as planned (and, if relevant, registered) have been explained.

## Data Availability

The data supporting the findings of this study could be made available upon reasonable request from the corresponding author.
